# Group Entropies: From Phase Space Geometry to Entropy Functionals via Group Theory

**DOI:** 10.3390/e20100804

**Published:** 2018-10-19

**Authors:** Henrik Jeldtoft Jensen, Piergiulio Tempesta

**Affiliations:** 1 Centre for Complexity Science and Department of Mathematics, Imperial College London, South Kensington Campus, London SW7 2AZ, UK; 2 Institute of Innovative Research, Tokyo Institute of Technology, 4259, Nagatsuta-cho, Yokohama 226-8502, Japan; 3 Departamento de Física Teórica, Universidad Complutense de Madrid, 28040 Madrid, Spain; 4 Instituto de Ciencias Matemáticas (ICMAT), 28049 Madrid, Spain

**Keywords:** generalised entropies, formal groups, phase space growth rate

## Abstract

The entropy of Boltzmann-Gibbs, as proved by Shannon and Khinchin, is based on four axioms, where the fourth one concerns additivity. The group theoretic entropies make use of formal group theory to replace this axiom with a more general composability axiom. As has been pointed out before, generalised entropies crucially depend on the number of allowed degrees of freedom *N*. The functional form of group entropies is restricted (though not uniquely determined) by assuming extensivity on the equal probability ensemble, which leads to classes of functionals corresponding to sub-exponential, exponential or super-exponential dependence of the phase space volume *W* on *N*. We review the ensuing entropies, discuss the composability axiom and explain why group entropies may be particularly relevant from an information-theoretical perspective.

## 1. Introduction

The aim of this paper is to discuss and review the construction of a class of entropies recently introduced, called group entropies [1,2,3,4]. We shall make several preliminary observations in order to ensure that our line of thinking is transparent.

In thermodynamics, according to Clausius the entropy ΔS=Q/T is defined macroscopically in terms of its change induced by a heat flow *Q* at temperature *T*. A connection to the microscopic world is obtained in statistical mechanics by Boltzmann’s expression

(1) $$S\left[p\right]=-\sum_{i=1}^{W}p_{i}\ln p_{i}=\ln W.$$

where the last equality is valid on the equal probability ensemble $p_{i}=1/W$ where $p_{i}$ is the probabilistic weight of state *i* and *W* denotes the number of available states. Hereafter, we assume $k_{B}=1$.

Jaynes made contact with information theory and pointed out that Boltzmann’s microcanonical and canonical ensembles can be viewed as the probabilistic weights that maximise the Boltzmann-Shannon entropy functional in Equation (1) under suitable constraints. The microcanonical ensemble is obtained when only the normalisation constraint is imposed, whereas the canonical ensemble arises when the normalisation and the average energy constraint are both assumed [5].

Here we will think of entropies in the spirit of information theory, i.e., functionals on probability space. Therefore the first three of the four Shannon-Khinchin axioms [6,7] are unobjectionable and the entropy of a system in the equal probability ensemble $p_{i}=1/W$ will be considered to a be a measure of uncertainty. It is then natural to assume that entropy in the equal probability ensemble is extensive (in the limit of a large number of particles). Namely, the more particles the more uncertain is the least biased ansatz $p_{i}=1/W$. We express this mathematically by saying that an entropy is *extensive* if, in the limit of large *N*, the entropy on the equal probability ensemble behaves as $S(p_{i}=1/W)=\lambda N$, N≫1. Hence we consider extensivity, defined in this way, to be a required property of the entropies we are going to consider. This is of course also done within the *q*-statistics framework [8]. It is also worth to recall that extensivity is a necessary condition for an entropy to play the role as a rate function in large deviation theory [9,10].

To try to keep our use of concepts clear and make a transparent distinction between extensivity and *additivity*, let us immediately mention, though we will elaborate below, that we consider an entropy to be additive, if, for two independent systems the entropy of the two systems considered as a whole is equal to the sum of the parts.

Once having established how the entropy of the entire system in the uniform ensemble scales with the number of particles (degrees of freedom), we need to make an equally important decision about composition of systems. Imagine a system that is obtained by merging two given systems *A* and *B* and assume that *A* and *B* are statistically *independent*. We start analysing this case not because we believe that real systems typically can be considered as collections of independent subsystems. Although in classical thermodynamics independence is often an excellent approximation, it is most likely not the case when dealing with complex systems. We consider the independent case for two reasons. First, one can always formally consider independent systems as constituting a whole and the entropy needs to be defined so it can handle this. Secondly, this requirement allows for important mathematical constraints on the entropy and, as explained in Section 2, establishes a link to group theory.

More precisely, since *A* and *B* are assumed to be independent, we can now either consider the cartesian product A×B of the states of the systems *A* and *B* as one system and compute the entropy of S(A×B), or we may as well first compute the entropy of the parts S(A) and S(B) and afterwards decide to consider A×B as a whole. We recall that entropies are functionals on probability space, which define a probabilistic weight for each of the microstates of a given system. For the independent combination considered here, we of course have that the microstates of A×B are given by the combined states (i,j) where *i* and *j* refer to the specific microstates of *A* and *B*, respectively. The independence ensures that the probability distributions describing *A*, *B* and A×B are related as $p_{i,j}^{A\times B}=p_{i}^{A}p_{J}^{B}$. So we need to ensure that the entropy functional computed using $p_{i,j}^{A\times B}$ is consistent with the expression obtained by computing first the functional on $p_{i}^{A}$ and $p_{j}^{B}$ and then combining the result. That is to say, we need a function ϕ(x,y) that takes care of the combination of the two independent systems *A* and *B* into one whole

(2) S(A×B)=ϕ(S(A),S(B)).

If the entropy is additive we have ϕ(x,y)=x+y. The relation in Equation (2) is of course basic in as much as it is a formality to consider the cartesian product A×B as a whole or as combined of two independent subsystems *A* and *B*. Equation (2) should therefore be satisfied for all possible choices of $p_{i}^{A}$ and $p_{j}^{B}$. In Section 2 below we discuss the properties of ϕ(x,y) in more detail. Here we just mention that Equation (2) ensures that in cases when the entire system can be considered to be a collection of subsystems, the entropy of a composed system S(A×B) depends on the entropy of A and the entropy of B of the component systems only, without the need for a microscopic description of them. Thus, in this way one can associate naturally the notion of entropy to a macroscopic system starting from the knowledge of its constituents. Complex systems are often defined as involving some degree of emergence, which is captured by the famous Aristotle’s quote “The whole is greater than the sum of the parts” (Methaphysics). A concrete explicit example of such situations was recently considered by introducing the so called pairing model [11] in which particles may combine to form new paired states which are entirely different from the single particle states. For a specific example think of hydrogen atoms. When two hydrogen atoms combine to form a hydrogen molecule, $H_{2}$ bound states are formed which cannot be reach within the cartesian product of the phase space of the two individual hydrogen atoms [12]. More generally, when dealing with complex systems the independent combination of subsystems will typically be different from the whole [13]. Let us by AB denote the system obtained by bringing the $N_{A}$ particles of system *A* together with the $N_{B}$ particles of system *B* and allowing for the two sets of particles to establish all possible interactions or interdependencies among the particles from *A* and those from *B*. In the example of the pairing model [11], AB will also contain new “paired states” among particles in *A* and particles in *B*. Therefore AB≠A×B since A×B consists only of the states that can be labelled as (i,j) where $i=1,\ldots ,W_{A}$ runs through all the states of system *A* and $j=1,\ldots ,W_{B}$ runs through all the states of system *B*. New *emergent* states formed by combining particles from *A* and *B* are not included in A×B.

To illustrate this point, think of system *A* and system *B* as consisting of a single hydrogen atom each. Then A×B is the set $A\times B=\{\left(r_{a},p_{a}\right),\left(r_{b},p_{b}\right)\}$ where $r_{i}$ and $p_{i}$ with i=a,b are the position and momenta of the hydrogen atom *A* or *B*. The combined system AB in contrast contains new emergent molecular states $H_{2}$ consisting of the hydrogen atom *A* bound together with the hydrogen atom *B*. We recall that the conventional description considers *H* and $H_{2}$ as two distinct ideal gases, introduces a chemical potential for each and minimises the Helmholtz free energy for the *H* and $H_{2}$ mixture, see e.g., Section 8.10 in [14]. In this way one does not need to handle super-exponentially fast growing phase spaces since $H_{2}$ is not considered a paired state of *H* atoms. The profound, though by now of course very familiar, concept of the chemical potential makes it possible to escape the combinatorial explosion in this specific case.

We require that the entropy evaluated on the equal probability ensemble for the fully interacting system satisfies (asymptotically) extensivity, i.e., that

(3) $$S\left(AB\right)=S\left(1/W\left(AB\right)\right)≃\lambda N_{AB}=\lambda \left(N_{A}+N_{B}\right)\;\mathrm{for}\;N_{A}\gg 1\;\mathrm{and}\;N_{B}\gg 1.$$

However, we cannot in general insist that

(4) S(AB)=ϕ(S(A),S(B))=S(A×B).

In the Boltzmann-Shannon case for which ϕ(x,y)=x+y the relation in Equation (4) holds when W(AB)=W(A)W(B), i.e., we have an exponential dependence $W\left(N\right)=k^{N}$. Below we’ll discuss in detail how the functional dependence W(N) of the total number of states on the number of particles will determine the properties of both the entropy and the composition law ϕ(x,y) and we’ll see that typically S(AB)≠S(A×B). When W(N) does not have an exponential behaviour, one either gets entropies equivalent to the Tsallis entropy, for sub-exponential algebraic dependence $W\left(N\right)=N^{a}$, or new group entropies for super-exponential phase space growth rates as, for instance, $W\left(N\right)=N^{\gamma N}$.

For complex systems, for which entropies are typically non-additive, the group entropies discussed here immediately suggest a measure of how complex a system is. Precisely because A×B≠AB for complex systems and therefore the entropy of the fully interdependent system AB will be different from the cartesian combination A×B, a measure of the essential emergent interdependence can be constructed as

(5) Δ(AB)=S(A×B)−S(AB)=ϕ(S(A),S(B))−S(AB).

This measure can be thought of as a possible generalisation of the usual mutual information and could perhaps be useful e.g., as an alternative to Tononi’s Integrated Information [15,16] as a measure that can quantify very entangled complex systems such as, say, consciousness. A thorough discussion of this complexity measure will appear in [17].

The remainder of the article is organised as follows. In Section 2, we present a brief and self-consistent introduction to group entropies. We explain in Section 3 how the phase space growth volume W(N) determines a specific group law that, in turn, enables us to characterise the functional form of allowed entropies and the rule for composing statistically independent systems. Precisely, we show that for a given function W(N) there exists a construction of dual entropies, a trace-form and a non-trace-form one, sharing over the uniform distribution the same composition law ϕ(x,y).

We relate the group entropies to existing entropies and discuss in Section 4 the probabilities $p_{i}$ derived by maximizing the entropy under constraints.

## 2. Basic Results on Group Entropies

In this section, we shall present a brief introduction to some basic aspects of the theory of group entropies. The mathematical apparatus will be kept to a minimum. For an more complete discussion, the reader is referred to the original papers [2,3,4,18]. We start out with the composition requirement in Equation (2). We need to require that (i) ϕ(x,y)=ϕ(y,x), since *A* and *B* are just labels that can obviously be interchanged. At the same time, we also require that the process of composition can be made in a associative way: (ii) ϕ(x,ϕ(y,z))=ϕ(ϕ(x,y),z). Finally, if system *B* is in a state of zero entropy, we wish that the entropy of the composed state A×B coincides with the entropy of *A*. In other words, (iii) Φ(x,0)=x. We shall say that an entropy satisfies the *composability axiom* if there exists a function ϕ(x,y) such that Equation (2) is satisfied, jointly with the previous properties of commutativity, associativity and composition with a zero-entropy state [1,3].

In order to ascertain the plausibility of the composability axiom, observe that, first of all, it is satisfied by Boltzmann’s entropy. It is a crucial requirement for possible thermodynamical applications. Indeed, it means that the entropy of a system composed of independent constituents depends on the macroscopical configuration of the constituents only, not on their microscopic properties. Therefore we can reconstruct the entropy of the whole system, in all possible configurations, just by knowing the entropy of its macroscopic parts. At the same time, property (2) is related to Einstein’s likelihood principle [19].

From a mathematical point of view, the composability axiom is equivalent to the requirement that ϕ(x,y) is a group law in the sense of formal group theory [20]. This is the origin of the group theoretical structure associated with the class of generalised entropies called group entropies [1,2,3]. To be precise, a *group entropy* is an entropic function satisfying the first three Shannon-Khinchin axioms and the composability axiom for all possible probability distributions. In this case the entropy is said to be composable in a strong sense. If an entropy is only composable on the uniform distribution, it is said to be weakly composable.

Thus, the connection between generalised entropies and group theory crucially relies on the composability axiom. Interestingly enough, the study of the algebraic structure defined by the family of power series ϕ(x,y) fulfilling the previous requirements has been developed in a completely different context, namely algebraic topology, during the second half of the past century. Here all we need to state is simply that a *one-dimensional formal group law* over a ring *R* [20] is a formal power series in two variables of the form

(6) $$\phi \left(x,y\right)=x+y+\sum_{ij}a_{ij}x^{i}y^{j}$$

That satisfies the properties (i)–(iii). The theory of formal groups was introduced by Bochner in the seminal paper [21] and developed in algebraic topology, analysis, and other branches of pure and applied mathematics by G. Faltings, S. P. Novikov, D. Quillen, J. P. Serre and many others [20,22]. For recent applications in number theory, see also [23,24].

A property crucial for the subsequent discussion is the following: given a one-dimensional formal group law ϕ(x,y) over a ring of characteristic zero, there exist a unique series $G\left(t\right)=t+\sum_{k=2}^{\infty }\beta_{k}t^{t}$ such that

(7) $$\phi \left(x,y\right)=G(G^{-1}\left(x\right)+G^{-1}\left(y\right)).$$

The relation between group entropies and formal group laws is therefore immediate. Indeed, a group entropy possesses a group law associated with it, expressed by a suitable function ϕ(x,y) of the form (6) which is responsible for the composition process for any possible choice of the probability distributions on *A* and *B*. A natural question is how to classify group entropies. To this aim, we recall that, generally speaking, we can distinguish between two large classes of entropy functions, the trace-form class and the non-trace-form one. In the first case, we shall deal with entropies that can be written as $S=\sum_{i}f\left(p_{i}\right)$ for a suitable one-variable function f(x). The prototype of this family is Boltzmann’s entropy. If an entropy cannot be written in this way, it is said to be a non-trace-form one. The most well-known example of a non-trace-form entropy is Rényi’s entropy. In this paper we shall focus on the following realizations of the two classes.

For the *trace-form class*, we shall analyse the general functional [3]

(8) $$S\left[p\right]=\sum_{i=1}^{W}p_{i}G\left(\ln\frac{1}{p_{i}}\right)$$

called the universal-group entropy (since it is related with the algebraic structure called Lazard’s universal formal group). Here G(t) is an arbitrary real analytic invertible function such that G(0)=0, $G^{\prime }\left(0\right)=1$.

For the *non-trace-form class* we shall consider the functional [3]

(9) $$S\left[p\right]=\frac{G\left(\ln(\sum_{i=1}^{W}p_{i}^{\alpha })\right)}{1-\alpha }$$

that has been called *Z*-entropy. Both families of entropies are assumed to satisfy the first three Shannon-Khinchin axioms for suitable choices of G(t). The main difference between the trace-form and the non-trace-form class is encoded in a theorem proved in [18], stating that the most general trace-form entropy satisfying Equation (2) is Tsallis entropy, with Botzmann’s entropy as an important special case. The infinitely many other possible trace-form entropies only fulfil the composition law Equation (2) on the restricted part of probability space consisting of uniform probabilities $p_{i,j}^{A\times B}=1/W_{A\times B}=1/\left(W_{A}W_{B}\right)=p_{i}^{A}p_{j}^{B}$. Therefore, these entropies are said to be weakly composable [3]. Instead, the non-trace-form entropy (9) is composable for any combination A×B of systems *A* and *B* with $p_{i,j}^{A\times B}=p_{i}^{A}p_{j}^{B}$.

## 3. From Phase Space Volume to Group Entropies

Extensivity and the dependence on the size of phase space have often played a role in the analysis of entropies. For the case of Tsallis entropy, the requirement of extensivity is used to determine the value of the parameter *q* [8,25]; the importance of the dependence of the entropy on the available number of microstates *W* was discussed in [26]. Here we describe how exploiting the relation between the number of microstates *W* and the number of particles *N* allows one to find the functional form of the group entropies, see [11,17]. For a discussion not assuming the composability requirement and hence nor the group structure, see [27,28].

We consider how the group-theoretic entropies deal with the three asymptotic dependencies of the phase space volume(I)Algebraic$W\left(N\right)=N^{a}$with$W^{-1}\left(t\right)=t^{\frac{1}{a}}$,(II)Exponential$W\left(N\right)=k^{N}$with$W^{-1}\left(t\right)=\frac{\ln t}{\ln k}$,(III)Super-exponential$W\left(N\right)=N^{\gamma N}$with$W^{-1}\left(t\right)=\exp\left[L\left(\frac{\ln t}{\gamma }\right)\right]$.

Here L(t) denotes the Lambert function.

Now we shall discuss how the extensivity requirement and the functional form of W(N) determine the function G(t), which in turn characterize the entropy according to formulae (8) and (9). Preliminary, before entering into technical details let us clarify how the present theory relates to previous investigation.

First, what could make the entropy non-additive? For the exponential case (II) we will find that the composition in Equation (2) corresponds to simple addition ϕ(x,y)=x+y. This is the traditional Boltzmann-Shannon case. All four S-K axioms, including the 4th additivity axiom, are satisfied and in accordance with the uniqueness theorem [6] we find $S\left[p\right]=-\sum_{i}p_{i}\ln p_{i}$. So, as one could expect, an exponential-type phase space volume is related with additivity and no essential emergence of interdependence among the components of the considered system. The situation turns out to be different for the cases (I) and (III) above. In both these cases W(AB)≠W(A×B)=W(A)W(B). In the sub-exponential case (I) the fully interdependent system AB has fewer states available than W(A)W(B). This situation is akin to how the Pauli principle prevents a set of fermions from occupying all possible combinations of single particle states. Instead, in case (III) the system AB has more states available than W(A)W(B), new collective states have emerged when *A* and *B* are combined [11].

Lieb and Yngvason has argued [29] that from standard classical thermodynamics, without any use of statistical mechanics, it follows that entropy must be additive and extensive. We recall that the fourth Shannon-Khichin [1] axiom assumes additivity and since the four SK axioms uniquely lead to the Boltzmann-Shannon functional form we can only be consistent with traditional thermodynamics if we remain within the Shannon-Khinchin axiomatic framework. This implies that only case (II) $W\left(N\right)=k^{N}$ is consistent with traditional thermodynamics. The two cases (I) $W\left(N\right)=N^{a}$ and $W\left(N\right)=N^{\gamma N}$ turns out not to be consistent with additivity, which takes one outside the framework of Boltzmann-Shannon-Khinchin and therefore in accordance with Lieb and Yngvason outside standard thermodynamics [8,30,31] i.e., we are naturally lead to the abstract conceptual framework of information theory. We wish to stress that group entropies represent measures of complexity by information geometric means [32] and can characterise limiting probability distributions by means of a maximum entropy procedure for systems where interdependence among its components makes W(N) deviate from the exponential form.

Stepping outside the SK framework can of course be done in multiple ways. One may simply decide to entirely give up on the 4th axiom and only assume the first three. This approach was considered in [26,27]. The group theoretic approach described here is of course related, however, importantly, it requires that a entropy must be defined in a way that allows the computation of the entropy of the independent combination A×B to be related in a consistent and unique way to the entropy of the parts *A* an *B*.

### 3.1. From W(N) to G(t)

We start from the requirement that the group entropy is extensive on the equal probability ensemble $p_{i}=1/W$, i.e., we require asymptotically for large *N*, and therefore large *W* (here we are assuming that *W*(*N*) is a monotonically increasing function of *N*) that

(10) $$S\left(p_{i}=\frac{1}{W}\right)=\lambda N.$$

We now consider separately the trace form case (8) and the non-trace form (9) one. For the first case, we have asymptotically

(11) $$S\left(\frac{1}{W}\right)=\sum_{i=1}^{W}\frac{1}{W}G\left(\ln\left(W\right)\right)\approx \lambda N.$$

Inverting the relation between *S* and *G*, which by Equation (11) amounts to inverting the relation between *G* and *N*, we obtain

(12) $$G\left(t\right)\approx \lambda W^{-1}\left[\exp\left(t\right)\right].$$

This is a consequence of the asymptotic extensivity. However, we also need G(t) to generate a group law, which requires G(0)=0 [1,3], so we adjust the expression for G(t) in Equation (12) accordingly and conclude

(13) $$G\left(t\right)=\lambda \{W^{-1}\left[\exp\left(t\right)\right]-W^{-1}\left(1\right)\}.$$

Assuming the non-trace form in Equation (9) when inverting Equation (10), and ensuring G(0)=0 leads to

(14) $$G\left(t\right)=\lambda \left(1-\alpha \right)\{W^{-1}\left[\exp\left(\frac{t}{1-\alpha }\right)\right]-W^{-1}\left(1\right)\}.$$

Assuming that *W*(*N*) is sufficiently regular, it is easy to see that the simple choice

$$\lambda =1/{\left(W^{-1}\right)}^{\prime }\left(1\right)$$

in both cases makes G of the form $G\left(t\right)=t+O\left(t^{2}\right)$.

From the expressions (13) and (14) we can now list the entropies corresponding to the three classes (I), (II) and (III) of phase space growth rates. A straightforward calculation gives the following results:

*Trace-form case*
  (I) Algebraic, $W\left(N\right)=N^{a}$:
    (15) $$\begin{array}{c} S[p]=a\sum_{i=1}^{W(N)}p_{i}\left[{\left(\frac{1}{p_{i}}\right)}^{\frac{1}{a}}-1\right] \end{array}$$
    (16) $$\begin{array}{c} =\frac{1}{q-1}\left(1-\sum_{i=1}^{W(N)}p_{i}^{q}\right). \end{array}$$
    To emphasize the relation with the Tsallis q-entropy, we have introduced q=1−1/a. Please note that the parameter *q* is determined by the exponent *a*, so it is controlled entirely by W(N).
  (II) Exponential, $W\left(N\right)=k^{N}$, k>0:
    (17) $$S\left[p\right]=\sum_{i=1}^{W(N)}p_{i}\ln\frac{1}{p_{i}}.$$
    This is of course the Boltzmann-Gibbs case.
  (III) Super-exponential, $W\left(N\right)=N^{\gamma N}$, γ>0:
    (18) $$S\left[p\right]=\sum_{i=1}^{W(N)}p_{i}\left\{\exp\left[L(-\frac{\ln p_{i}}{\gamma })\right]-1\right\}.$$
*Non-trace-form case*
  (I) Algebraic, $W\left(N\right)=N^{a}$:
    (19) $$S\left[p\right]=\left\{\exp\left[\frac{\ln(\sum_{i=1}^{W(N)}p_{i}^{\alpha })}{a(1-\alpha )}\right]-1\right\}.$$
  (II) Exponential, $W\left(N\right)=k^{N}$:
    (20) $$S\left[p\right]=\frac{\ln(\sum_{i=1}^{W(N)}p_{i}^{\alpha })}{1-\alpha }.$$
    This is of course the Rényi entropy.
  (III) Super-exponential, $W\left(N\right)=N^{\gamma N}$:
    (21) $$S\left[p\right]=\left\{\exp\left[L\left(\frac{\ln\sum_{i=1}^{W(N)}p_{i}^{\alpha }}{\gamma (1-\alpha )}\right)\right]-1\right\}.$$
    This entropy was recently studied in relation to a simple model in which the components can form emergent paired states in addition to the combination of single particle states [11].

### 3.2. The Composition Law ϕ(x,y)

We now derive the composition law introduced in Equation (2) above. The composition is given in terms of the function G(t) as in [3,4] according to the relations

*Trace-form case* [33]
  (22) $$\phi \left(x,y\right)=G[G^{-1}\left(x\right)+G^{-1}\left(y\right)].$$
*Non-trace-form case*
  (23) $$\phi \left(x,y\right)=\frac{1}{1-\alpha }G\left[G^{-1}\left(\left(1-\alpha \right)x\right)+G^{-1}\left(\left(1-\alpha \right)y\right)\right].$$

When we express ϕ(x,y) directly in terms of the phase space volume W(N) by use of Equations (13) and (14) we arrive at the following expression valid for both trace and non-trace forms

(24) $$\phi \left(x,y\right)=\lambda \left\{W^{-1}\left[W\left(\frac{x}{\lambda }+W^{-1}\left(1\right)\right)W\left(\frac{y}{\lambda }+W^{-1}\left(1\right)\right)\right]-W^{-1}\left(1\right)\right\}$$

where $\lambda =1/{\left(W^{-1}\right)}^{\prime }\left(1\right)$.

To obtain from Equation (24) specific realisations of ϕ(x,y) for the three phase space growth rates, we substitute the appropriate expressions for W(N) and $W^{-1}\left(t\right)$ and obtain the following results.

(I) Algebraic, $W\left(N\right)=N^{a}$:
  (25) $$\phi \left(x,y\right)=x+y+\frac{1}{\lambda }xy=x+y+\left(1-q\right)xy.$$
  The case of Tsallis and and Sharma-Mittal entropies (see also [32] for new examples).
(II) Exponential, $W\left(N\right)=k^{N}$:
  (26) ϕ(x,y)=x+y.
  The Boltzmann and Rényi case.
(III) Super-exponential, $W\left(N\right)=N^{\gamma N}$:
  (27) $$\phi \left(x,y\right)=\lambda \left\{\exp\left[L(\left(1+\frac{x}{\lambda }\right)\ln\left(1+\frac{x}{\lambda }\right)+\left(1+\frac{y}{\lambda }\right)\ln\left(1+\frac{y}{\lambda }\right))\right]-1\right\}$$
  For examples of models relevant to this growth rate and composition law see [11,28].

## 4. Maximum Entropy Ensembles

Let us now consider the probability distributions derived from the group entropies by maximizing them under very simple constraints. As usual, we shall introduce the constraints by means of Lagrange multiplies and analyse the functional

(28) $$J\left[p\right]=S\left[p\right]-\sum_{n=1}^{M}\lambda_{n}g_{n}\left[p\right],$$

with *M* constraints expressed by the functionals $g_{n}\left[p\right]$. Traditionally, one uses the first constraint to control the normalization

(29) $$g_{1}\left[p\right]=\sum_{i=1}^{W}p_{i}-1$$

and the second one to determine the average of some observable *E*. In physics, this observable is typically the average of the systems energy $\bar{E}=\langle E\rangle -E_{0}$ measured from the ground state level $E_{0}$

(30) $$g_{2}\left[p\right]=\sum_{i=1}^{W}\left(p_{i}\left(E_{i}-E_{0}\right)-\bar{E}\right).$$

With these two constraints, from the extremal condition $\delta J/\delta p_{i}=0$ we obtain

(31) $$\frac{\delta S[p]}{\delta p_{i}}=\lambda_{1}+\lambda_{2}\left(\Delta E_{i}-\bar{E}\right).$$

Here $\Delta E_{i}=E_{i}-E_{0}$.

### 4.1. Trace-Form Entropies

The derivatives of S[p] for the three trace-form ones in Equations (16)–(18) are

(I) Algebraic – $W\left(N\right)=N^{a}$:
  (32) $$\frac{\delta S}{\delta p_{i}}=a\left(1-\frac{1}{a}\right){\left(\frac{1}{p_{i}}\right)}^{\frac{1}{a}}.$$
(II) Exponential – $W\left(N\right)=k^{N}$:
  (33) $$\frac{\delta S}{\delta p_{i}}=\left(\ln\frac{1}{p_{i}}-1\right).$$
(III) Super-exponential – $W\left(N\right)=N^{\gamma N}$:
  (34) $$\frac{\delta S}{\delta p_{i}}=\exp\left[L(X_{i})\right]-\frac{1}{\gamma }\frac{1}{1+L(X_{i})}-1,\;\mathrm{with}\;X_{i}=\frac{1}{\gamma }\ln\frac{1}{p_{i}}.$$

The functional form of $\delta S/\delta p_{i}$ in Equations (32) and (33) allows us to solve Equation (31) to express $p_{i}$ as

(I) Algebraic – $W\left(N\right)=N^{a}$:
  (35) $$p_{i}=\frac{{\left[1+\beta \left(\Delta E_{i}-\bar{E}\right)\right]}^{-a}}{Z},$$
  where formally $\beta =\lambda_{2}/\lambda_{1}$ and $Z=\sum_{i}{\left[1+\beta \left(\Delta E_{i}-\bar{E}\right)\right]}^{-a}$. These probabilistic weights correspond to Tsallis’s q-exponentials.
(II) Exponential – $W\left(N\right)=k^{N}$:
  (36) $$p_{i}=\frac{\exp[-\beta \left(\Delta E_{i}-\bar{E}\right)]}{Z}$$
  where formally $\beta =\lambda_{2}/\lambda_{1}$ and $Z=\sum_{i}\exp\left[-\beta \left(\Delta E_{i}-\bar{E}\right)\right]$. As expected, we have re-derived the Boltzmann weights starting from the trace form of the group entropies and exponential phase space growth rates.

The transcendental nature of the expression for $\delta S/\delta p_{i}$ in Equation (34) seems to prevent one from deriving a closed formula for $p_{i}$ in the case of super-exponential phase space growth rate $W\left(N\right)=N^{\gamma N}$, having assumed the trace-form (weakly composable) expression (18) for the entropy. We shall see below that the situation is different when starting from non-trace-form entropies.

### 4.2. Non-Trace-Form Entropies

The form of the entropies for the non-trace case given in Equations (19)–(21) all lead to the same functional expression as when starting from the trace-form algebraic case in Equation (35), namely

(37) $$p_{i}=\frac{{\left[1+\beta \left(\Delta E_{i}-\bar{E}\right)\right]}^{\frac{1}{1-\alpha }}}{Z}$$

where formally $\beta =\lambda_{2}/\lambda_{1}$ and $Z=\sum_{i}{\left[1+\beta \left(\Delta E_{i}-\bar{E}\right)\right]}^{\frac{1}{1-\alpha }}$. This expression for $p_{i}$ is reminiscent of the Tsallis q-exponential.

## 5. Discussion

We have seen that the group theoretic entropies offer a systematic classification of entropies according to how the phase space grows with number of particles. The formalism allows for a systematic generalisation of a statistical mechanics description to non-exponential phase spaces and reduces to the standard Boltzmann-Gibbs picture when W(N) is an exponential. A new measure of complexity, see Equations (5) and (24), determined by the phase space volume’s dependence on system size follows right away.

We wish to point out that group entropies represent an interesting new tool in information geometry, since they can be used to construct Riemannian structures in statistical manifolds via suitable divergences (or relative entropies) [34] associated with them. This has been proved in [32] for Z-entropies and in [35] for the universal-group entropy. Also, a quantum version of these entropies can be used to define a family of entanglement measures for spin chains [1]. Work is in progress along all these lines.
